# A novel necroptosis-related LncRNA signature for prediction of prognosis and therapeutic responses of head and neck squamous cell carcinoma

**DOI:** 10.3389/fphar.2022.963072

**Published:** 2022-08-09

**Authors:** Zilu Meng, Wenhan Yang, Lei Zhu, Wanyu Liu, Yudong Wang

**Affiliations:** Department of Maxillofacial Surgery, The First Affiliated Hospital of Guangdong Pharmaceutical University, Guangzhou, China

**Keywords:** head and neck squamous cell carcinoma, lncRNA, necroptosis, prognosis, multi-omics, chemotherapy, immunotherapy

## Abstract

**Background:** Long non-coding RNAs (lncRNAs) play an essential role in the occurrence and prognosis of tumors, and it has great potential as biomarkers of tumors. However, the roles of Necroptosis-related lncRNA (NRLs) in Head and neck squamous cell carcinoma (HNSCC) remain elusive.

**Methods:** We comprehensively analyzed the gene expression and clinical information of 964 HNSCC in four cohorts. LASSO regression was utilized to construct a necroptosis-related lncRNA prognosis signature (NLPS). We used univariate and multivariate regression to assess the independent prognostic value of NLPS. Based on the optimal cut-off, patients were divided into high- and low-risk groups. In addition, the immune profile, multi-omics alteration, and pharmacological landscape of NLPS were further revealed.

**Results:** A total of 21 NRLs associated with survival were identified by univariate regression in four cohorts. We constructed and validated a best prognostic model (NLPS). Compared to the low-risk group, patients in the high group demonstrated a more dismal prognosis. After adjusting for clinical features by multivariate analysis, NLPS still displayed independent prognostic value. Additionally, further analysis found that patients in the low-risk group showed more abundant immune cell infiltration and immunotherapy response. In contrast, patients in the high-risk group were more sensitive to multiple chemotherapeutic agents.

**Conclusion:** As a promising tool, the establishment of NLPS provides guidance and assistance in the clinical management and personalized treatment of HNSCC.

## Introduction

Head and neck squamous cell carcinoma (HNSCC) accounts for the sixth most common cancer globally, with 890,000 new cases per year and 450,000 deaths per year (Chow, 2020). Despite the continuous progress in the treatment of HNSCC (including surgery, immunotherapy, radiotherapy, and chemotherapy, et al.), its morbidity and mortality are still gradually increasing, with the gross 5-years survival rate of only 60% (Torre et al., 2015). In addition, because of the high risk of recurrence and metastasis, the survival rate of advanced HNSCC patients was only 35% (Chauhan et al., 2015). In recent years, the tumor node metastasis (TNM) staging in the American Joint Commission on Cancer (AJCC) grading system has become an important basis for the clinical management and treatment of HNSCC patients (Amin et al., 2017). However, this approach has several problems, such as different clinical behaviors between patients with the same TNM stage, different treatment responses, and substantial variability in clinical outcomes (Kim et al., 2016; Carnielli et al., 2018). Thus, clinicians are unable to provide treatment based on clinicopathological characteristics alone. To better stratify patients and identify high-risk patients, developing a robust signature for predicting HNSCC patient outcomes is necessary.

As a programmed form of cell death, necroptosis triggers an inflammatory response by increasing the permeability of the cell surface, leading to the release of cellular contents (Beretta and Zaffaroni, 2022). Due to the strong proinflammatory features, necroptosis plays an important role in inflammatory diseases and viral defense processes, including viral myocarditis, and Alzheimer’s disease (Zhao et al., 2022; Zhou et al., 2022). Recently, the researchers found that necroptosis inhibited tumor progression and metastasis by regulating programmed tumor cell death in various solid tumors (Beretta and Zaffaroni, 2022). Necroptosis has been used as a novel biomarker to guide the treatment of cancer patients in clinical practice (Chen et al., 2022; Luo et al., 2022; Zhan et al., 2022). For example, Luo et al. have precisely predicted the prognosis and treatment benefit of colorectal cancer patients by developing necrosis-related molecular characteristics (Chen et al., 2022). Zhang et al. found that necrosis-related genes affect the immune status and overall survival of breast cancer patients (Zhang et al., 2022). However, research correlating necrotic genes with patient outcomes in head and neck tumors is still lacking.

Long non-coding RNA (LncRNA) is a type of non-coding RNA molecules and has more than 200 nucleotides. Despite the limited coding capacity of lncRNAs, it still occupies an essential role in regulating cancer cell behavior (differentiation, migration, and apoptosis) and cancer progression (Huang et al., 2017). For instance, the lncRNA molecule HOTTIP inhibited the proliferation of HNSCC cells by up-regulating the TLR5/NF-κB pathway (Jiang H. et al., 2022). Additionally, the interaction of lnc-POP1-1 with MCM5 promotes the resistance of HNSCC cells to cisplatin (Jiang Y. et al., 2022). Therefore, previous studies have shown that lncRNAs play an important role in HNSCC (Jiang H. et al., 2022; Jiang Y. et al., 2022). However, necrosis-related lncRNAs are lacking to be studied in HCSSS, and the molecular mechanisms by which they affect tumors need to be further explored.

In our study, we comprehensively analyzed gene expression and clinical information of HNSCC in the TCGA database and constructed a necroptosis-driven lncRNA prognosis signature (NLPS). Subsequently, the prognosis value of NLPS was further evaluated in three published cohorts. According to the NLPS score, patients were divided into different risk groups and exhibited significantly different survival characteristics. Overall, the establishment of NLPS promotes the development of stratified management and personalized treatment of HNSCC patients in clinical practices.

## Methods

### Data collection and processing

The gene expression and clinical information of 964 HNSCC samples were downloaded from the Cancer Genome Atlas (TCGA, https://portal.gdc.cancer.gov/) and Gene Expression Omnibus (GEO, http://www.ncbi.nlm.nih.gov/geo/) databases, which were stored in the TCGA-HNSC (*n* = 494), GSE41613 (*n* = 97), GSE42743 (*n* = 103), and GSE65858 (*n* = 270) datasets. The inclusion and exclusion criteria of samples were as followings: 1) primary HNSCC; 2) Completed overall survival (OS) information; 3) Preoperative chemotherapy or chemoradiotherapy were not utilized to patients. Additionally, we obtained the HumanMethylation450 array and somatic mutation data from the TCGA GDC website (https://portal.gdc.cancer.gov/). In FireBrowse website (http://firebrowse.org/), copy number variation (CNV) data were retrieved and processed by the GISTIC2.0 algorithm (Reich et al., 2006). To further explore the treatment benefit of HNSCC, the drug sensitivity information and gene expression profile were obtained from PRISM (https://www.theprismlab.org/), CTRP (https://portals.broadinstitute.org/ctrp.v2.1/), and CCEL (https://sites.broadinstitute.org/ccle/) datasets. The baseline characteristics of patients are summarized in Supplementary Table S1.

### Necroptosis gene set

Based on the MSigDB website (https://www.gsea-msigdb.org/gsea/index.jsp) and previous literatures in the PubMed database (Petanidis et al., 2020; Wu et al., 2022), 117 Necroptosis-related genes were selected. The gene set was displayed in Supplementary Table S2.

### Necroptosis-related lncRNA

Refer to previous studies of Liu et al. (Liu et al., 2021a), we constructed a pipeline to identify the Necroptosis-related lncRNAs (NRLs). The details of the pipeline were as followings: 1) The correlation of necrotic-related genes with each lncRNA was calculated. The necrotic-related genes were ranked in descending order based on the correlation values. 2) The ranked gene list was subjected to enrichment analysis by the ‘GSEA’ package, and the Necroptosis enrichment score of each lncRNA was calculated. 3) The lncRNAs were identified as NRLs according to the following two criteria: the false discovery rate (FDR) < 0.05 and necroptosis enrichment score (NES) > 0.995.

### Univariate cox regression

In order to screen NRLs with prognosis value, univariate cox regression was applied. Due to differences in sequencing platforms and centers, the same gene can exhibit opposite prognostic significance in different cohorts. Although they showed strong prognostic significance in single cohort, they cannot be used as a biomarker for judging prognosis. Based on the above reason, we screened genes whose absolute hazard ratio (HR) > 1 in more than 50% of the cohort and maintained the same orientation as stable prognostic genes.

### LASSO regression

With the development of high-throughput sequencing technology, researchers have developed a variety of powerful machine learning algorithms, which were widely used in the exploration process of bioinformatics. In the process of linear regression analysis, the correlation between variables causes multicollinearity, which will distort the results of the model. In order to screen variables more strictly and further reduce or eliminate multicollinearity, the LASSO regression algorithm was utilized, which removes confounding variables by penalizing the coefficients of variables (Lockhart et al., 2014). To construct a Necroptosis-driven lncRNA prognosis signature (NLPS), ten cross-validations were performed in the ‘glmnet’ R package and the lambda value was selected when the partial likelihood deviation reached a minimum. HNSCC patients were divided into high- and low-risk groups according to the optimal cut-off point. Finally, we successfully constructed the NLPS model in the TCGA-HNSC cohort and validated it in the GSE41613, GSE42743, and GSE65858 cohorts.

### Evaluation of the NLPS

We explored the prognosis values of the high- and low-risk groups through the Kaplan-Meier analysis. Receiver operating characteristic (ROC), and calibration curves were utilized to assess the accuracy of NLPS, which were calculated by the ‘timeROC’ and ‘rms’ R packages, respectively. In addition, we explored the independent prognosis value of NPLS after adjusting for variables (age, gender, and AJCC et al.) by univariate and multivariate logistic regression analysis, which were presented by forest plot.

### Gene set enrichment analysis and gene set variation analysis

The prognostic stratification of patients in the clinic was often due to the differences in underlying molecular mechanisms, and it is necessary to explore the enriched functions and pathways of gene profiles in the two subgroups. Since gene set enrichment analysis (GSEA) was sensitive to biological processes, it was used to explore the enrichment state of genes clustered in different pathways (Hung et al., 2012). In order to prepare the gene list, we performed differential analysis (count file form TCGA-HNSC cohort) of patients in the high- and low-risk groups by the ‘DESeq2’ R package and ranked all genes in descending order according to log2FoldChange (log2FC). Subsequently, the ranked gene list was performed to GO and KEGG (Molecular Signatures Database, version: c5. go.v7.5.1. symbols.gmt and c2. cp.kegg.v7.5. symbols.gmt) enrichment analyses in the ‘enrichplot’ R package. In addition, we also used gene set variation analysis (GSVA) in the ‘GSVA’ R package to further explore the significantly changed functions and pathways between the two groups patients.

### Evaluation of immune infiltration and immunotherapy response

Immunotherapy as a novel treatment modality was widely used in multiple solid tumors in clinical practice (Ma et al., 2020). Therefore, ssGSEA was utilized to calculate the abundance of 28 immune cells infiltrating of the two groups patients (Subramanian et al., 2005). Likewise, we also compared the expression of immune co-stimulatory and co-inhibitory molecules between patients in the two subgroups. Immunophenoscore (IPS) is a comprehensive score to determine immunogenicity, including 27 immune molecules from B7-CD28 superfamily, TNF superfamily, and other molecules (CD27, CD28 et al.) (Charoentong et al., 2017). The ‘deconvo_tme’ function in the ‘IOBR’ R package was used to calculate the IPS score of each HNSCC patient (Zeng et al., 2021). The abundance of immune cells partly reflects the effect of patients receiving immunotherapy, so we further analyzed the response rate of patients in different groups to multiple immunotherapies. As an unsupervised clustering approach algorithm, the Subclass Mapping (SubMap, https://cloud.genepattern.org/) revealed common subtypes between independent cohorts (Liu et al., 2021b). In our study, we assessed the response to CTLA-4 and PD-1 therapy by calculating the similarity of patients in the high- and low-risk groups to melanoma patients receiving immunotherapy. In addition, tumor immune dysfunction and exclusion (TIDE, http://tide.dfci.harvard.edu/) was utilized to predict the response to immune checkpoint inhibitors (ICIs) of each patient.

### Mapping of mutation landscapes

The mutational signature of each patient was extracted by the ‘maftools’ R package, and the tumor mutational burden (TMB) was calculated. TMB was defined as the number of somatic, indel, coding, and base substitution mutations per Mb of genome examined (Chalmers et al., 2017), which includes the single nucleotide variations (SNV) and insertion deletion (INDEL). We defined the top 30 of the genes with mutation frequency as high-frequency mutated genes (FMGs) by calculating the mutation frequency of each gene. We mapped the mutational landscape of FMGs and calculated their differences in mutation frequency between high- and low-risk groups. As a previous study (Allgauer et al., 2018), TMB will affect the abundance of neoantigens to some extent. Therefore, we compared the difference in neoantigen load between the two groups, which was downloaded from the TCGA website. In order to further explore the prognosis value of FMGs, univariate and multivariate logistic regression analyses were applied to calculate the odds ratio (OR) values of each FMG.

### Copy number variation and methylation-driven genes

In order to explore the copy number variation (CNV) of patients in the two groups, the GISTIC 2.0 algorithm was used. Likewise, the prognostic value of the ten chromosomal segments with the highest CNV was explored by univariate and multivariate analysis.

Based on previous research (Jung et al., 2019), we identified methylation-driven genes (MDGs) in the high- and low-risk groups by the ‘MethylMix’ R package. Finally, we also compared the differences of the methylation abundances, expression levels, and methylation frequencies between the two groups.

### Explore potential chemotherapeutic agents

We collected the gene expression and drug sensitivity information from the CCLE, CTRP, and PRISM websites. Based on a previous study pipeline (Yang et al., 2021), cell lines were divided into high- and low-risk groups by calculating NLPS scores. Subsequently, we determined potential therapeutic drugs for patients in different risk groups by comparing the area under the ROC curve (AUC) differences of drugs between high- and low-risk cell lines. Lower AUC indicates higher sensitivity. To evaluate the accuracy of analysis process, we calculated the relationship between cisplatin sensitivity and the high and low groups of TAP1, ATM, and LCN2. Based on the previous researches (Matassa et al., 2016; Zhang et al., 2017; Huang et al., 2019), low expression of ATM et al. would increase the sensitivity of tumor cells to cisplatin.

### Statistical analysis

All data processing, calculation, analysis, and visualization were performed in the R software (version 4.1.2). The comparisons between continuous variables were calculated by the T-test and Wilcoxon rank-sum test. The chi-square test was utilized to make comparisons between categorical variables. KM curve with the log-rank test was utilized to survival analysis. The FDR value was calculated by the Benjamin–Hochberg (BH) method. *p* < 0.05 was regarded as statistically significant. Statistical tests were all two sides.

## Results

### Construction of the NLPS model

**FIGURE 1:**
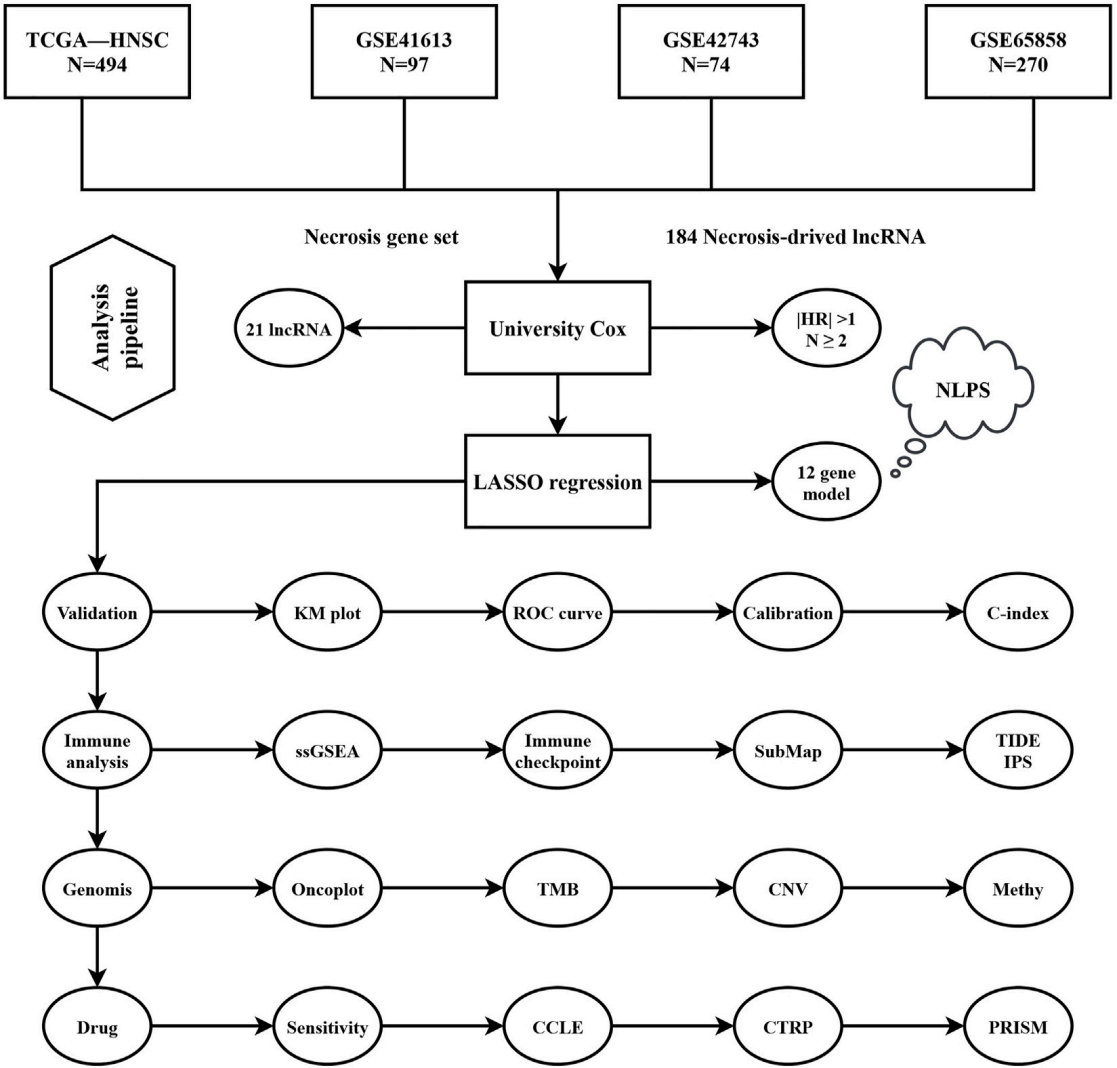
Flowchart of analysis procedure.

The analysis pipeline of our study was displayed in Figure 1. Based on the mRNA and lncRNA gene expression, a total of 184 necrose-related lncRNAs (NRLs) were identified. The prognosis values of NRLs were analyzed by univariate COX regression.

**FIGURE 2:**
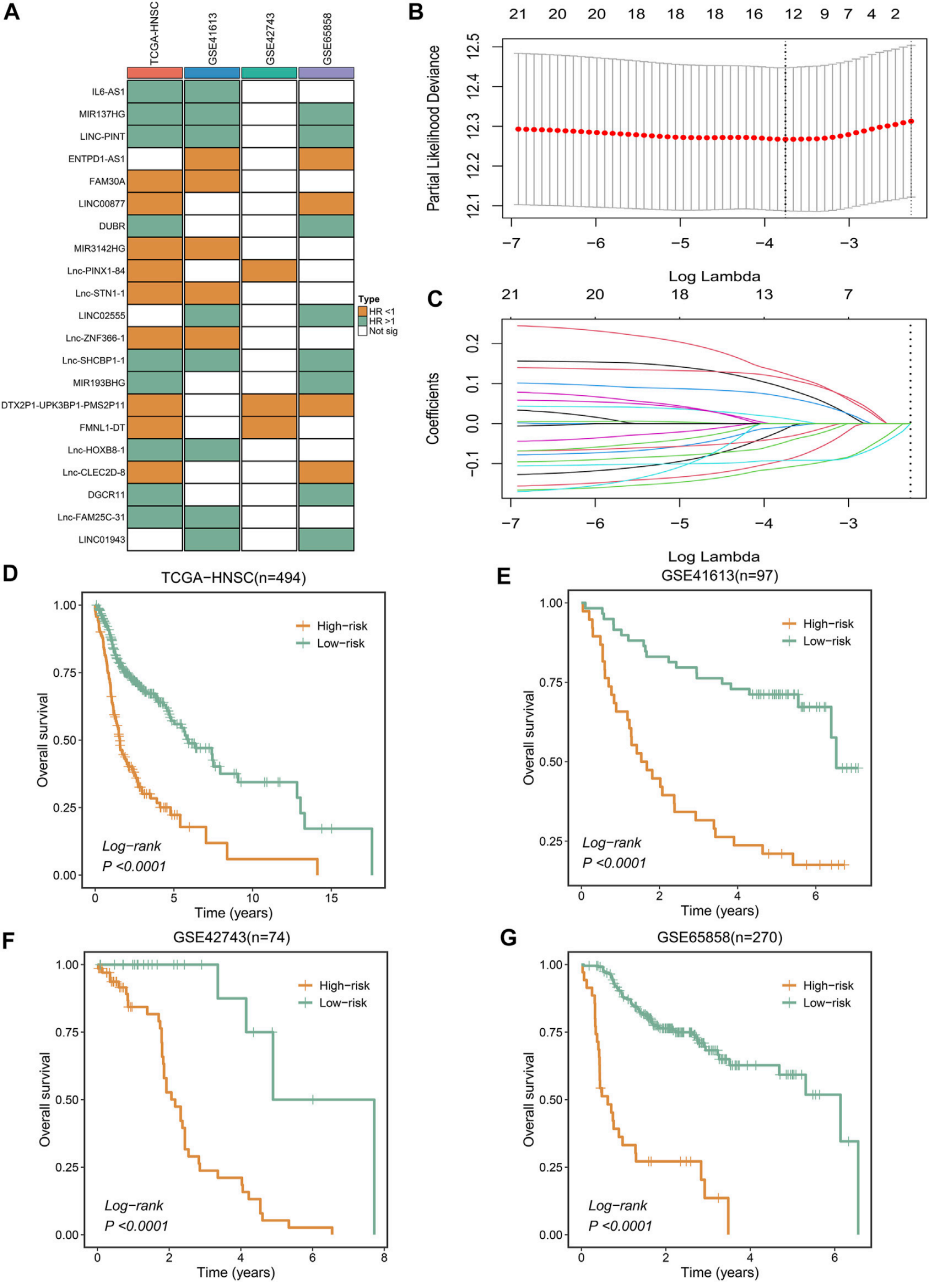
Integrative construction of a robust signature. **(A)** Discovery of 21 consensus prognosis genes from four independent multi-center cohorts. **(B,C)** Least absolute shrinkage and selection operator (LASSO) logistic regression algorithm to screen of gene associated with prognostic. **(D–G)** Kaplan–Meier curves of OS according to the NLPS in TCGA-HNSC **(D)**, GSE41613 **(E)**, GSE42743 **(F)**, GSE65858 **(G)**.

We found that only 21 NRLs have independent prognostic significance in over 50% of cohorts (Figure 2A). Subsequently, the expression information of the 21 stable prognosis genes was analyzed using LASSO regression algorithm in the TCGA-HNSC cohort (Figures 2B,C). We successfully constructed the optimal model when the lambda was minimum, including 12 NRLs (Supplementary Table S2). Through the NLPS model we calculate the risk score for each patient and divide them into high- (n = 142) and low-risk (n = 352) subgroups according to the optimal cut-off point. Compared with the low-risk group, high-risk patients showed more dismal prognosis in the TCGA-HNSC cohort (Figure 2D). Notably, our findings were similarly demonstrated in three validation cohorts (Figures 2E–G).

### Highly prognosis value of NLPS

**FIGURE 3:**
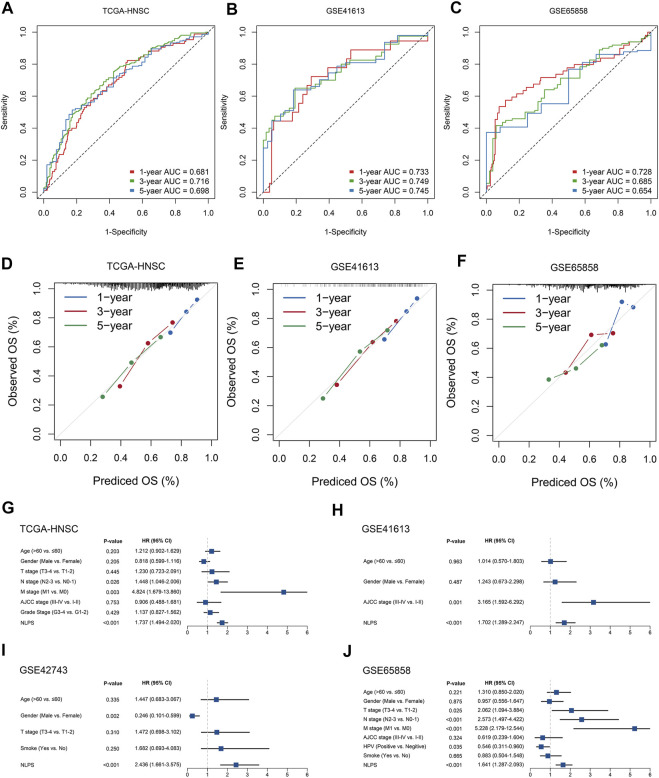
The evaluation of NLPS. **(A–C)** Kaplan-Meier curves of OS between the two groups in TCGA-HNSC **(A)**, GSE41613 **(B)**, and GSE65858 **(C)**. **(D–F)** Calibration plots were used to compare the actual probabilities and the predicted probabilities of OS in the three cohorts. **(G–J)** Multivariate COX regression analysis of the risk score.

To assess the robustness of the NLPS model, we calculated the area under the ROC curve (AUC) with OS at 1/3/5 years, which were respectively 0.681/0.716/0.698 (TCGA-HNSC, Figure 3A), 0.733/0.749/0.745 (GSE41613, Figure 3B), 0.728/0.685/0.654 (GSE65858, Figure 3C), and 0.650/0.681/0.655 (GSE42743, Supplementary Figure S1). Likewise, the calibration curve further proved the high accuracy of the NLPS model in the four cohorts (Figures 3D–F and Supplementary Figure S2). In addition, NLPS had higher C-index in all cohort, and respectively were 0.667 (95% confidence interval (CI): 0.629–0.705, TCGA-HNSC), 0.695 (95% CI: 0.621–0.770, GSE41613), 0.651 (95% CI: 0.564–0.738, GSE42743), and 0.653 (95% CI: 0.586–0.721, GSE65858). As we know, clinical characteristics occupy an important role in the clinical management of patients, and whether the model can be independent of clinical characteristics is the key to evaluating its efficacy. As illustrated in (Figures 3G–J), multivariate regression analysis indicated that NLPS remained an independent prognostic factor after adjusting for clinical characteristics (AJCC stage, T, N, M, HPV, smoke et al.)

### Abundance of immune infiltrates was higher in the low-risk group patients

**FIGURE 4:**
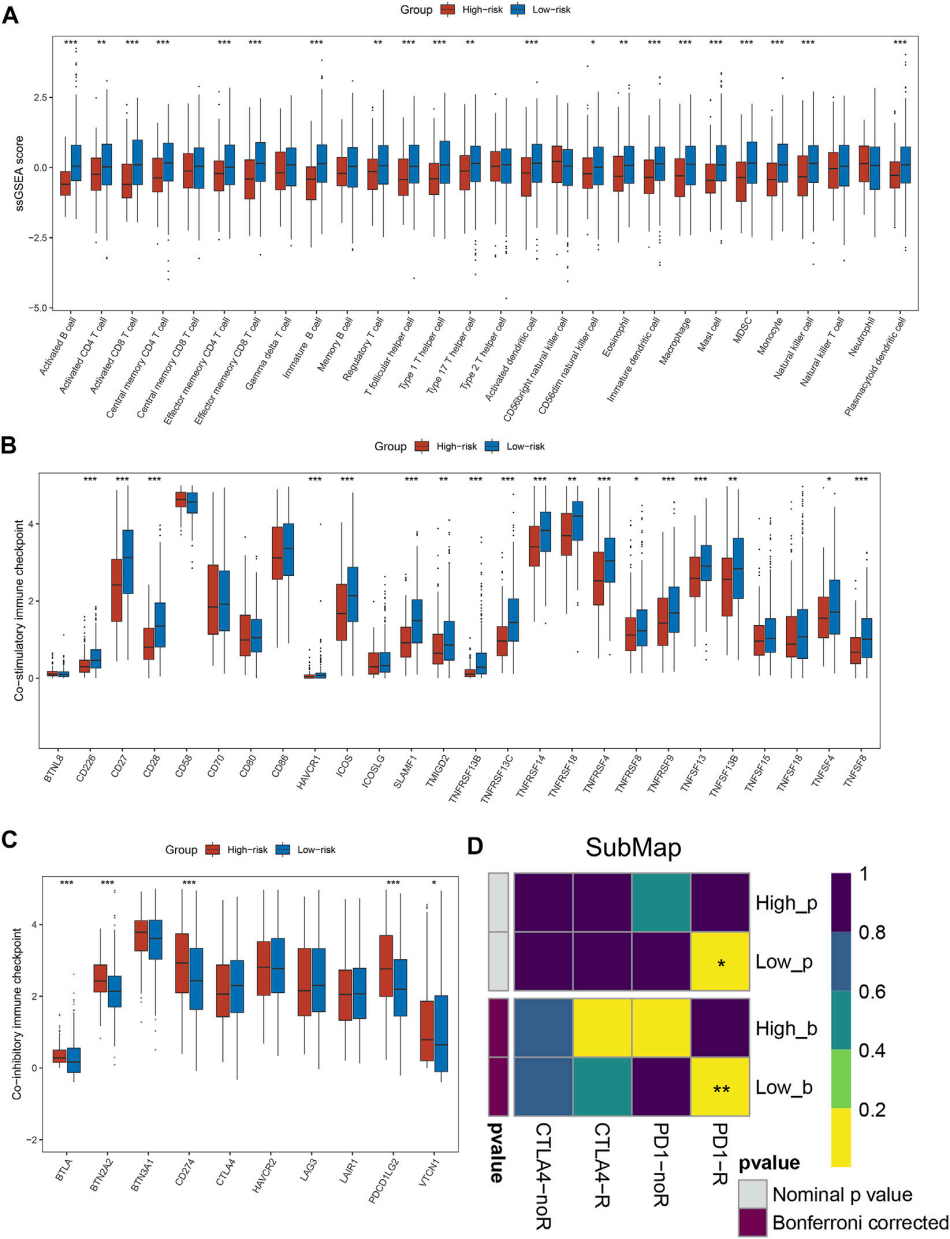
Immune infiltration analysis and prediction of immunotherapy response in the two groups. **(A)** The abundance of 28 immune cells between the two groups. **(B,C)** Comparison of immune co-stimulatory **(B)** factor and immune co-inhibitory **(C)** between the two groups. **(D)** SubMap algorithm evaluated the expression similarity between the two phenotypes and the patients with different immunotherapy responses. *p < 0.05, **p < 0.01, ***p < 0.001, ****p < 0.0001.

**FIGURE 5:**
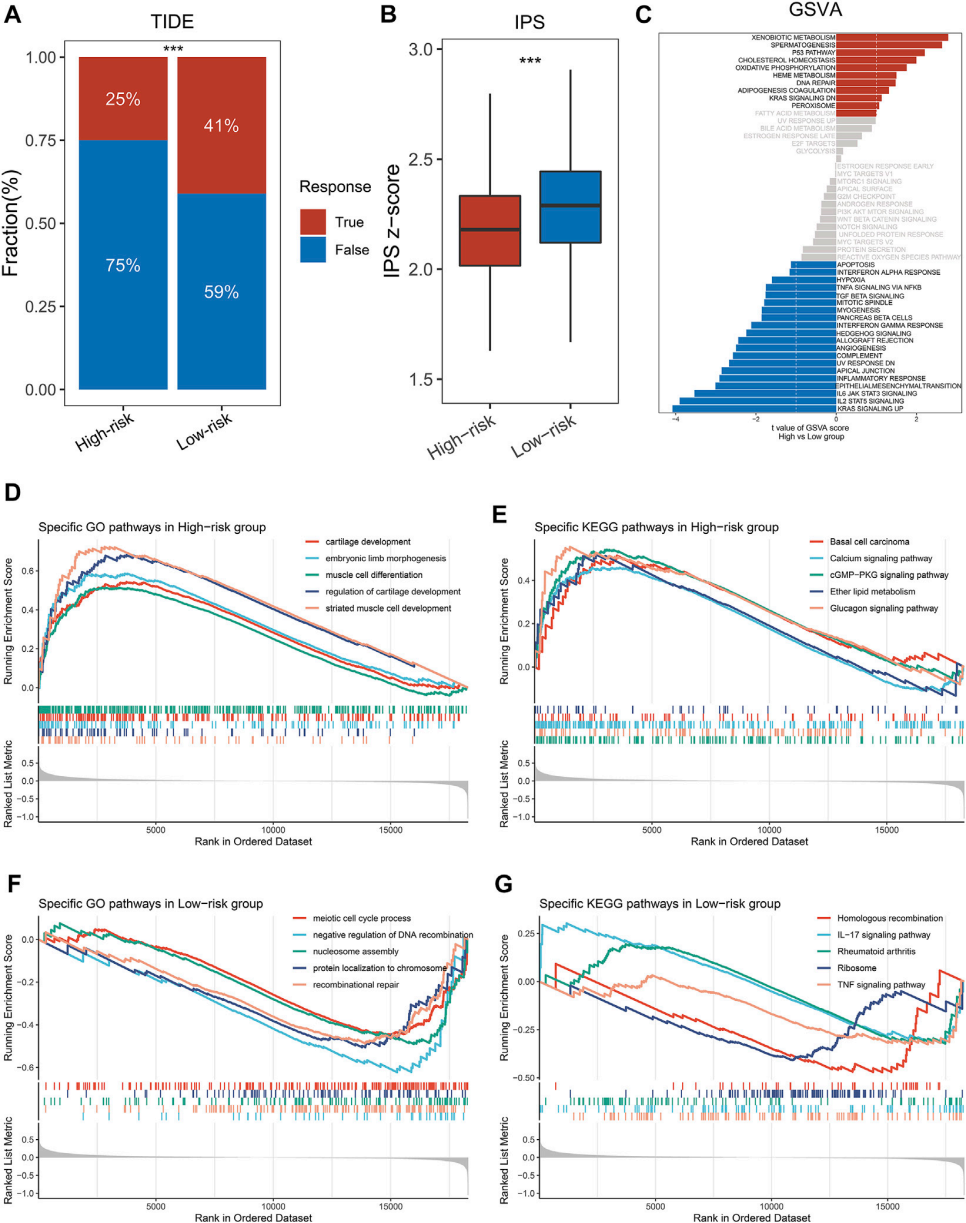
Exploring the potential mechanisms of NLPS. **(A)** TIDE was utilized to predict the response of immune checkpoint inhibitors in high- and low-risk groups. **(B)** The difference of IPS score between the high- and low-risk. **(C)** GSVA enrichment analysis. **(D–G)** The top five GO terms and KEGG pathways in the two groups. *p < 0.05, **p < 0.01, ***p < 0.001, ****p < 0.0001.

By the ssGSEA method, we calculated the infiltration abundance of 28 immune cells. As detailed in Figure 4A, patients in the low-risk groups exhibited more abundant immune infiltration than the high-risk group, especially activated CD4 T cell, activated CD8 T cell, central memory CD8 T cell, and effector memory CD8 T cell. To more accurately assess the abundance of immune molecules, we further compared the expression abundance of immune co-stimulatory and immune co-inhibitory molecules between the two groups patients. As expected, patients in the low-risk group exhibited higher immune co-stimulatory molecules (CD226, CD27, CD28, TNFSF8, SLAMF1 et al., Figure 4B) and lower levels of immune co-inhibitory molecules (BTLA, BTN2A2, CD274, VTCN1 et al., Figure 4C). In addition, the SubMap algorithm was utilized to compare the response of immunotherapy between the two groups, and the low-risk group patients were more sensitive to PD-1 treatment than the high-risk group (Figure 4D). TIDE and IPS analysis found that patients in the low-risk group had higher response rates to ICIs and IPS scores (Figures 5A,B), which further proved that the low-risk group patients had more abundant immune infiltration and could benefit more from immunotherapy.

### Patients in the high-risk group had stronger invasive characteristics

We explored significant differences in the GO terms and KEGG pathways between the two groups by the GSEA algorithm. As displayed in Figures 5D,E, patients in the high-risk group were significantly enriched in invasive- and developmental-related pathways, such as cartilage development, muscle cell differentiation, regulation of cartilage development, basal cell carcinoma, calcium signaling pathway et al. In contrast, patients in the high-risk group were significantly enriched in repair- and inflammatory-related pathways, such as IL-17 signaling pathway, rheumatoid arthritis, recombinational repair, meiotic cell cycle process et al. (Figures 5F,G). Notably, GSVA enrichment analysis showed that patients in the two subgroups were significantly enriched in different functions and pathways (Figure 5C). The high-risk group patients were significantly enriched in metabolic- and developmental-related pathways, such as xenobiotic metabolism, oxidative phosphorylation, spermatogenesis, heme metabolism et al. The high-risk group patients were significantly enriched in inflammatory-related pathways, such as TGF-β signaling, IL-2 STAT signaling, IL-6 JAK STAT signaling, mitotic spindle et al. Based on the above results, we found that the GSEA and GSVA analyses exhibited the same trend.

### Higher mutation frequencies exhibited in the high-risk group

**FIGURE 6:**
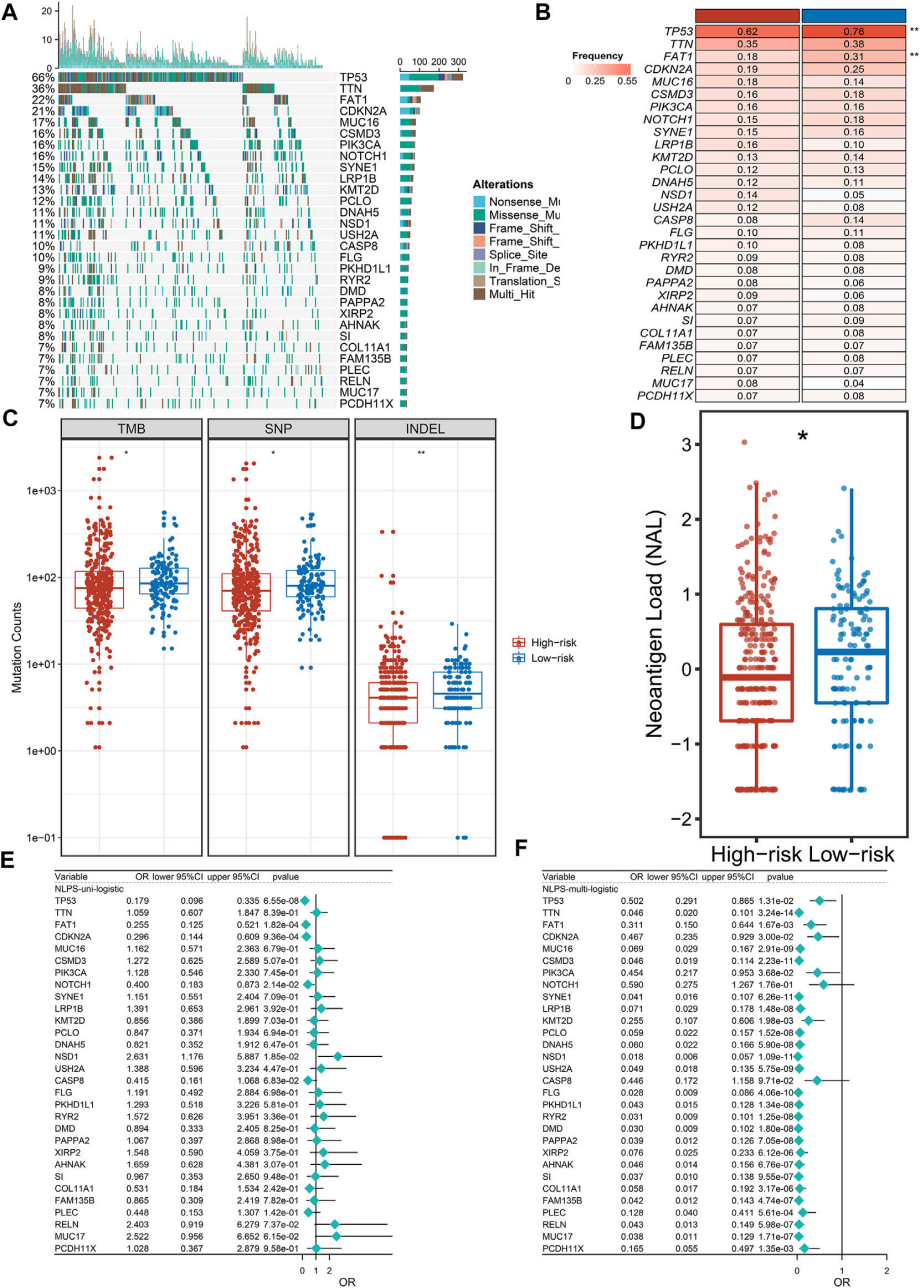
Genomic landscape of NLPS. **(A,B)** Mutational landscape **(A)** and frequency **(B)** of the top 30 FMGs between two groups. **(C)** The amount of TMB, SNP, and INDEL between two groups. **(D)** The difference of NAL between the high- and low-risk. **(E,F)** Correlation between the mutation status of top 30 FMGs and NLPS. *p < 0.05, **p < 0.01, ***p < 0.001, ****p < 0.0001.

Initially, we calculated the mutation frequency of each gene and defined the genes with the top 30 mutation frequencies as high-frequency mutation genes (FMGs), which was displayed in waterfall (Figure 6A). To further analyze the mutation status of the two groups of patients, we compared the mutation differences of FMGs between high- and low-risk group patients. As showcased in Figure 6B, patients in the low-risk group had higher mutation frequencies than high-risk patients, especially TP53 and FAT1. Subsequently, we compared the TMB of the two groups by boxplots. Patients in the low-risk group had higher TMB, either SNP or IMDEL (Figure 6C). TMB is the key to tumor neoantigen production. Unsurprisingly, patients in the low-risk group also had higher neoantigen loads (Figure 6D). Additionally, we further explore the prognostic value of the mutation status of FMGs. By univariate and multivariate regression analysis, we found that TP53 and FAT1 were independent prognostic factors even after adjustment for common clinical variables (Figures 6E,F).

### Differences in copy number variation and methylation-driven gene between the two groups

**FIGURE 7:**
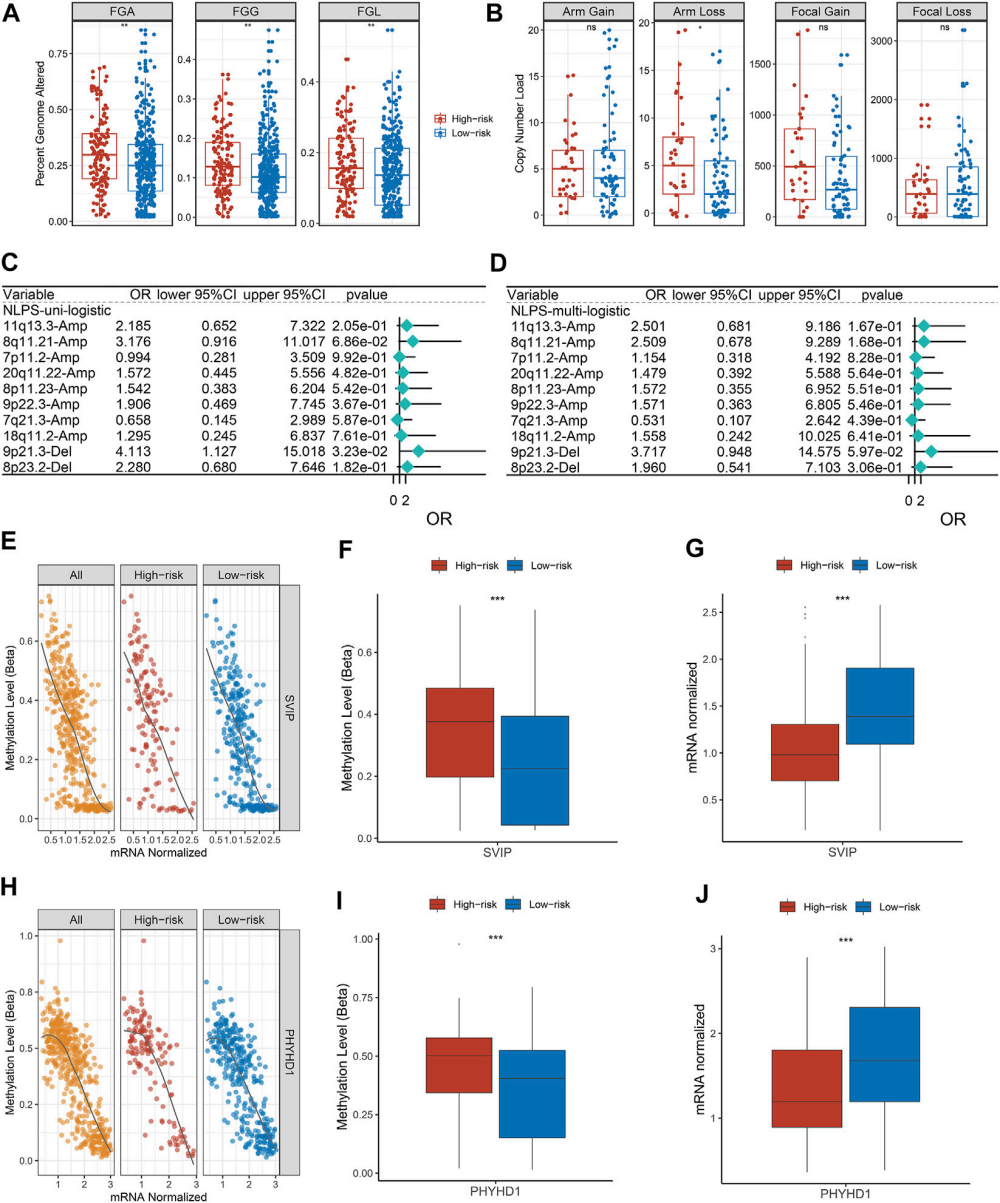
Copy number variation and methylation-driven genes. **(A)** Distributions of a fraction of genome alteration, gain and lost between two groups. **(B)** Distributions of arm gain, arm loss, focal gain, and focal loss. **(E,H)** Correlation between gene expression and methylation of SVIP **(E)** and PHYHD1 **(H)**. **(F,G)** The differences of SVIP methylation **(F)** and mRNA expression **(G)**. **(I,J)** The differences of SVIP methylation **(I)** and mRNA expression **(J)**. *p < 0.05, **p < 0.01, ***p < 0.001, ****p < 0.0001.

We further explored the copy number variation (CNV) and found that FGA, FGG, FGL, and arm loss in the high-risk group were significantly higher (Figure 7A). Although there was no significant difference in arm gain, focal gain, and focal loss between the two groups, the high-risk group still showed a higher trend (Figure 7B). Subsequently, we identified the top 10 chromosomal segments with the highest CNV and assessed their prognostic value by univariate and multivariate regression. As displayed in Figures 7C,D, the CNV status of most chromosomal segments was risk factor, but only 9p21.3-Del segments had independent prognostic significance. As one of the most well-known epigenetic mechanisms in tumor epigenetics, DNA methylation plays an important role in the progression of various solid tumors. Therefore, we further performed an association analysis of the methylation profiles and gene expression profiles in HNSCC patients. The methylation levels and transcriptome levels of SVIY and PHYHD1 were significantly negatively correlated, which were identified as MDGs (Figures 7E,H). Notably, compared with patients in the low-risk group, the methylation level (Figures 7F,I) and methylation frequency (Supplementary Figure S3A,B) of MDGs in the high-risk group were higher, while the transcriptome level was significantly lower (Figures 7G,J).

### High-risk patients more sensitive to chemotherapy drugs

**FIGURE 8:**
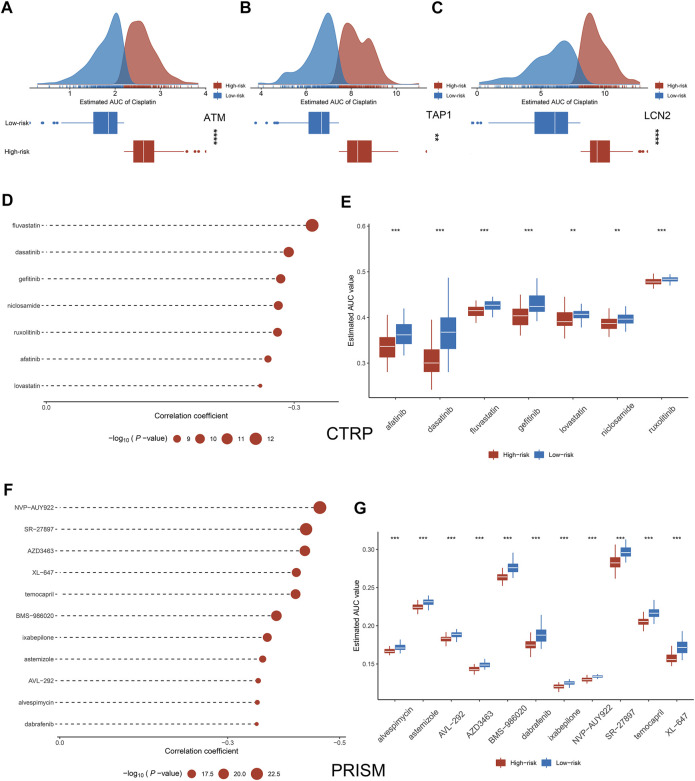
Exploration of potential therapeutic drugs. **(A–C)** Comparison of sensitivity to cisplatin in high and low groups of ATM **(A)**, TAP1 **(B)**, and LCN2 **(C)**. **(D–G)** Drugs sensitive to high-risk group cell lines were identified from CTRP and PRISM. *p < 0.05, **p < 0.01, ***p < 0.001, ****p < 0.0001.

Previous studies have shown that low expression of LCN2, ATM, and ATP1 increases the sensitivity of tumor cells to cisplatin (Matassa et al., 2016; Zhang et al., 2017; Huang et al., 2019). Based on the gene expression data and drug susceptibility information of the cell lines, our analysis reached the same conclusion, which further demonstrates the accuracy of our analytical pipeline (Figures 8A–C). Subsequently, seven drugs sensitive to high-risk group patients were identified from the CTRP, including afatinib, dasatinib, fluvastain, gefitinib, lovastatin, niclosamide, and ruxolitinib (Figures 8D,E). Likewise, a total of 11 drugs sensitive to high-risk group patients were identified from the PRISM database, such as XL-647, alvespimycin, astenmizole, AVL-292 et al. (Figures 8F,G).

## Discussion

Head and neck squamous cell carcinoma (HNSCC) has high histopathological heterogeneity and molecular heterogeneity, which poses a great obstacle to the clinical treatment and management of patients and leads to high mortality (Fujima et al., 2019; Chow, 2020). TNM staging alone as an indicator of clinical management and treatment has been unable to adapt to the personalized management and treatment of patients in the clinic. Therefore, the development of biomarkers that can predict the prognosis and treatment benefit of HNSCC patients is necessary. With the advancement of high-throughput sequencing technology and the development of bioinformatics, researchers have more deeply decoded HNSCC and derived a large number of biomarkers (Zhan et al., 2022; Zhang et al., 2022). These biomarkers covered a wide range of biological functions, including inflammation, pyroptosis, immunity, and hypoxia et al. (Han et al., 2022; Li et al., 2022; Lu and Jia, 2022; Zhu et al., 2022). In recent years, necroptosis played an important role in the development and progression of tumors (Zhang et al., 2022; Zhou et al., 2022). However, the mechanism of necroptosis-related lncRNAs in HNSCC remains to be further explored.

In our study, 184 necrose-related lncRNAs (NRLs) were identified through our established pipeline. Based on the univariate and LASSO regression analysis, a necroptosis-driven lncRNA prognosis signature (NLPS) was established and validated in four cohorts, which demonstrating the high accuracy of NLPS. As is well known, the ability to stratify the prognostic and underlying biological characteristics of patients is a testament to the efficiency of the model (Kaseb et al., 2011). KM and ROC curves indicate that NLPS can accurately predict the prognosis of patients in different centers, which suggests that NLPS has clinical generalizability. Prognostic differences between patients were often due to their underlying molecular mechanisms and characteristics (Guo et al., 2022). Based on the GSEA and GSVA algorithms, we further explore the biological characteristics of patients in the high- and low-risk groups. Patients in the high-risk group were significantly enriched in invasive- and developmental-related pathways, such as muscle cell differentiation, which may be the source of the dismal prognosis of high-risk group patients. Lei et al. reported that the differentiation of myocytes promoted the proliferation and invasion of tumor cells (Lei et al., 2018). In contrast, patients in the low-risk group showed good prognosis due to their potent anti-inflammatory and immune function. Immune checkpoint inhibitors (ICIs) displayed great potential as an emerging therapeutic modality in a variety of solid tumors (Korde et al., 2022). Immune cell abundance underlies the response of cancer patients to treatment with ICIs (Liu et al., 2022). Compared to the high-risk group patients, patients in the low-risk group demonstrated a higher abundance of immune cells. Based on the above findings, we inferred that patients in the low-risk group were more sensitive to immunotherapy and proved our conjecture by TIDE and SubMap algorithms.

With the development of genome sequencing technology, researchers have performed deeper decoding of tumors (Gambera et al., 2018). A previous report suggested that genomic mutations affect tumor patient prognosis and immunotherapy (Witkiewicz et al., 2015). We found that patients in the low-risk group had a higher tumor mutation burden (TMB), especially in TP53 and FAP1. The higher TMB of patients in the low-risk group indicated a higher response rate to immunotherapy, which further validated our conclusion. The higher TMB of patients in the low-risk group indicated that they have higher response rate to immunotherapy, which further validated our conclusion. Subsequently, Subsequently, we found that patients in the high-risk group had higher CNV than patients in the low-risk group. As a variation of DNA structure, CNV is an important cause of disease progression and phenotypic variation in humans (Jeng et al., 2013). The higher FGA, FGG, and FGL may be the reason why patients in the high-risk group displayed a dismal prognosis. Additionally, an increasing number of researches have displayed that methylation driver genes (MDGs) occupy an important role in the epigenetic regulation of HNSCC (Nakagawa et al., 2021). We found a significant inverse correlation between methylation and transcriptome levels for both SVIP and PHYHD1, which were identified as MDGs. Compared to the low-risk group, patients in the high-risk group had higher methylation frequencies. We hypothesize that methylation of SVIP and PHYHD1 may contribute to dismal prognosis in HNSCC patients. Due to the current lack of studies correlating the methylation status of SVIP and PHYHD1 with the prognosis of HNSCC, our findings may provide new ideas for the treatment of HNSCC. Overall, the homogeneity among transcriptional, mutational, CNV, and methylation profiles results further illustrates that NLPS has great clinical application value as a tool to predict the prognosis of HNSCC patients.

With the diversification of treatment modalities in the clinic, overdiagnosis and treatment become an urgent problem, which not only allows patients to bear expensive costs but also increases the risk of treatment complications (Zubiri et al., 2021). Different from the previous study focusing on the prognosis of HNSCC, we further predicted sensitivity to chemotherapeutic drugs in the two groups patients. Although NLPS can accurately identify high-risk patients by stratifying patients, how to perform beneficial treatment for high-risk patients is an urgent clinical problem. According to previous findings, low expression of ATM, TAP1 and LCN2 will increase the sensitivity of tumor cells to cisplatin (Matassa et al., 2016; Zhang et al., 2017; Huang et al., 2019), which is the same as our study conclusion and further illustrates the accuracy of our method. Based on CTRP and PRISM databases, we identified a total of 18 sensitive chemotherapeutic agents for high-risk patients, including afatinib, dasatinib, et al. These drugs were widely used in a variety of solid tumors, but their use in HNSCC has rarely been reported (Laviolette et al., 2017). Therefore, the establishment of NLPS provides guidance for the identification and treatment of high-risk patients in HNSCC. As an important finding of this study, this has important implications for the treatment of HNSCC patients.

Our study comprehensively evaluated the prognostic characteristics and therapeutic benefits of HNSCC. Although our study needs to be validated in subsequent clinical trials, studies with large samples and multiple centers fully illustrate the high precision and clinical generalizability of NLPS.

## Conclusion

In conclusion, we comprehensively analyzed the genetic and clinical information of HNSCC and established a stable model (NLPS), which can accurately predict the prognosis and clinical benefit of HNSCC patients and displayed robust efficacy in different cohorts. In addition, we further revealed the immune profile, multi-omics alteration, and pharmacological landscape of NLPS, which has significantly meaningful in predicting the benefit of immunotherapy and chemotherapy. Overall, the establishment of NLPS provides guidance and assistance in the clinical management and personalized treatment of HNSCC.

## Data Availability

The datasets presented in this study can be found in online repositories. The names of the repository/repositories and accession number(s) can be found in the article/Supplementary Material.
