# Supplementary material for: A novel necroptosis-related LncRNA signature for prediction of prognosis and therapeutic responses of head and neck squamous cell carcinoma
Source: Front Pharmacol. 2022 Aug 9;13:963072. doi: 10.3389/fphar.2022.963072 (PMC9395581; doi:10.3389/fphar.2022.963072)
Supplement: Supplementary file 1 [file DataSheet2.docx]

**Supplementary Figures**

- **Figure S1**
- **Figure S2**
- **Figure S3**


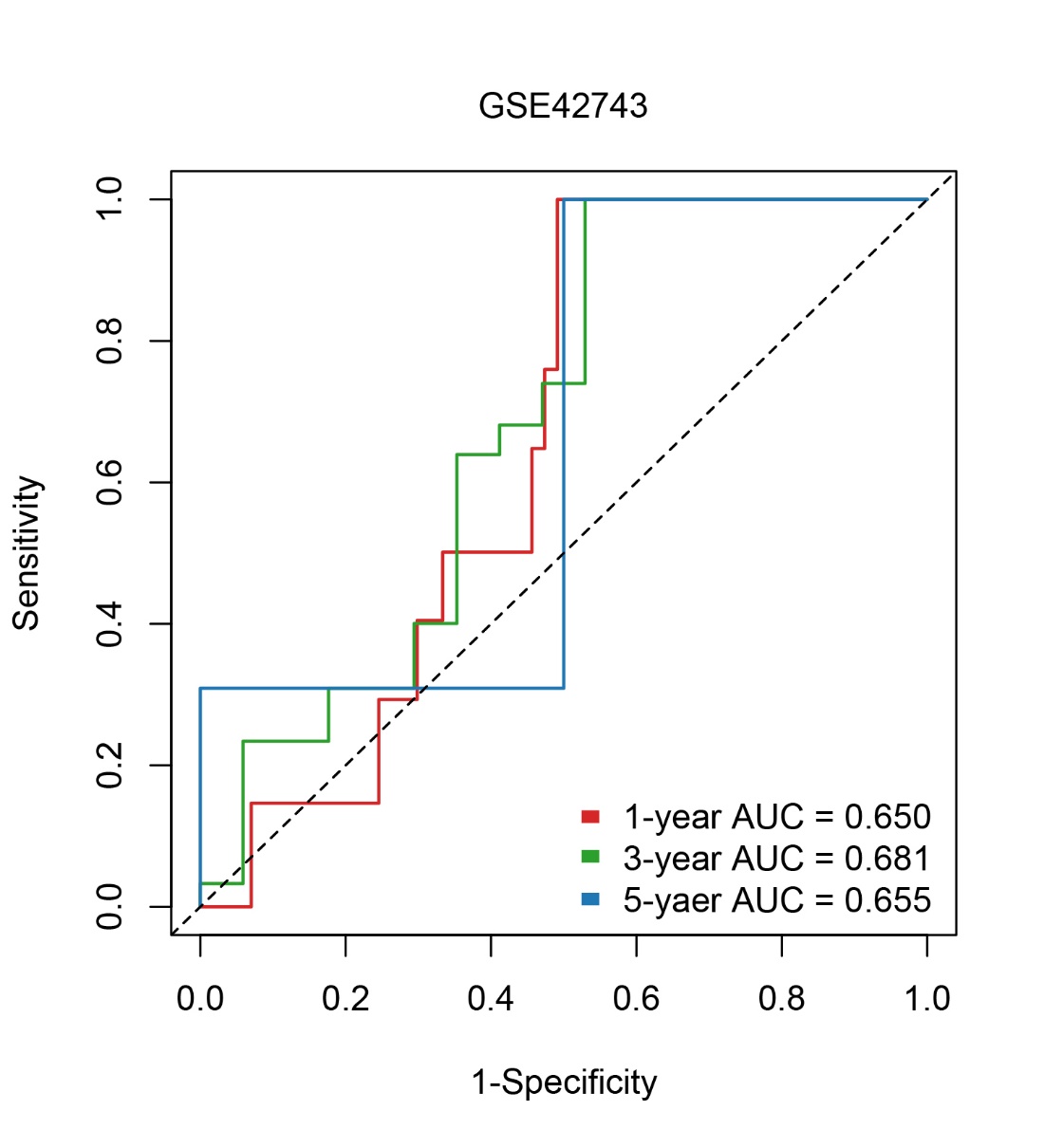


**Figure S1. Kaplan-Meier curves of OS between the two groups in the GSE42743 cohorts.**


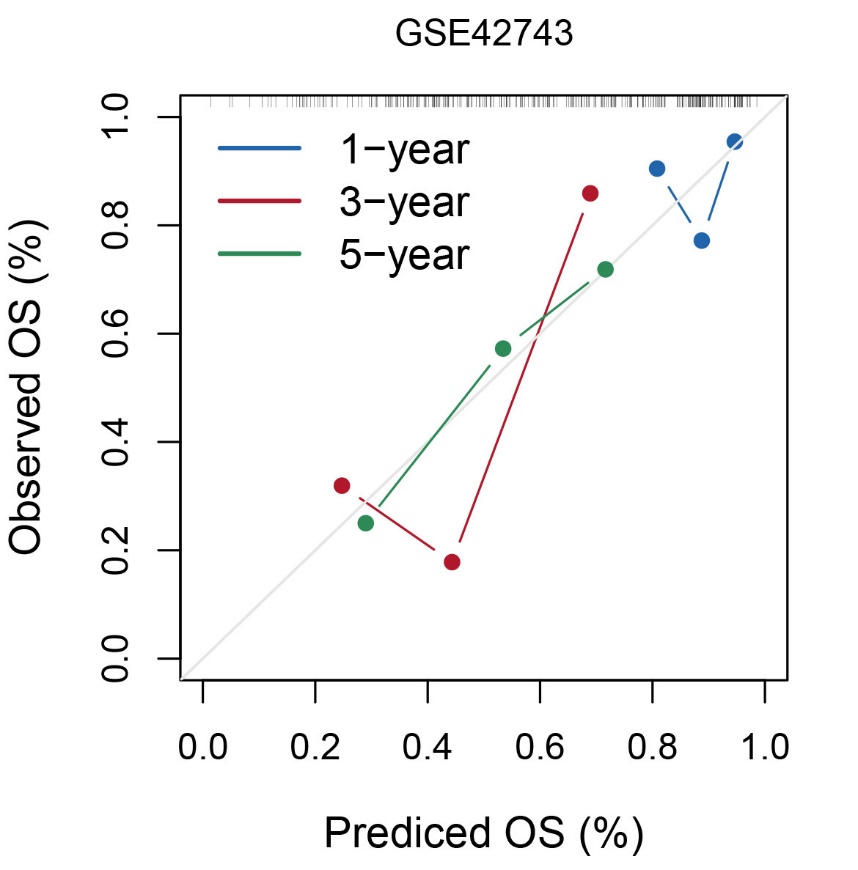


**Figure S2**. **Calibration plots of OS in the GSE42743 cohorts.**


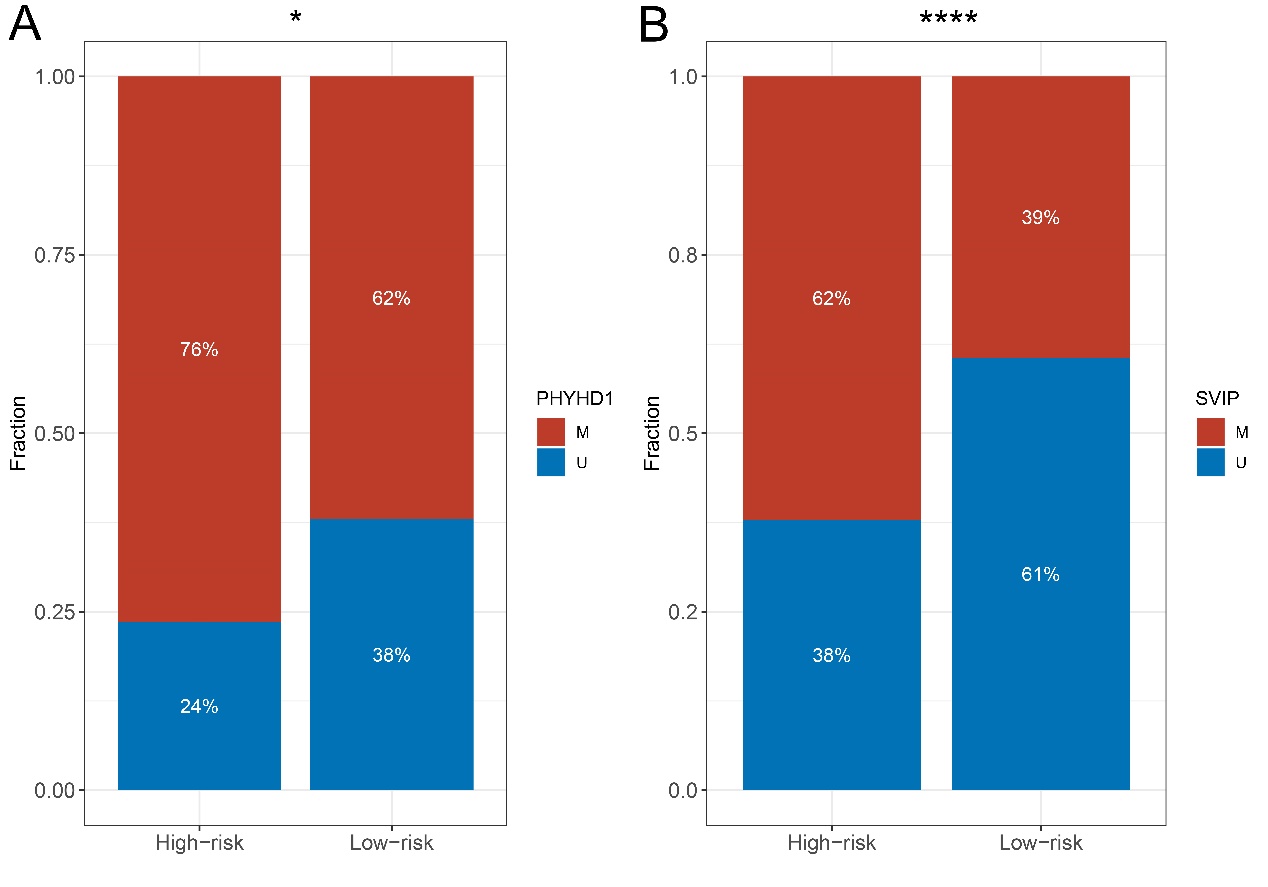


**Figure S3**. **The mutation frequency of SVIP (A) and PHYHD1 (B) between the two groups.**
